# Sleep and activity patterns in autism

**DOI:** 10.1177/13623613251413538

**Published:** 2026-02-05

**Authors:** J Dylan Weissenkampen, Arpita Ghorai, Thaise NR Carneiro, Maria Fasolino, Brielle N Gehringer, Maya Rajan, Holly C Dow, Shriya Kunatharaju, Till Roenneberg, Ronnie Sebro, Daniel J Rader, Brendan T Keenan, Laura Almasy, Edward S Brodkin, Maja Bućan

**Affiliations:** 1 University of Pennsylvania, USA; 2 Ludwig-Maximilians-University Munich, Germany; 3 Mayo Clinic, USA; 4 Children’s Hospital of Philadelphia, USA

**Keywords:** actimetry, autism, circadian behavior, physical activity, sleep

## Abstract

Autism is a heritable neurodevelopmental condition marked by impaired social interaction, repetitive behavior, and co-occurring conditions. Sleep disturbances are common in autism. This study uses low-cost wearable devices to compare sleep, physical activity, and circadian behavior in autistic adults and their non-autistic relatives. We recruited 318 autistic individuals and 130 family members, collecting accelerometer data over 3 weeks (8249 days). Using a data-driven approach, we identified actimetry-derived features associated with autism. We examined 308 traits using the elastic net algorithm and linear mixed effects regressions. We identified 52 actimetry measures associated with autism (area under the curve: 0.812; confidence interval: 0.761–0.862), validated in a test set (area under the curve: 0.756; confidence interval: 0.700–0.813). Both mean and day-to-day variability in several measures (e.g., time spent sedentary, total light physical activity) were associated with autism. In autistic individuals, reduced physical activity during wake was more strongly associated with shorter sleep time than in non-autistic relatives (likelihood ratio: 41.6; p = 1.13e−10). Reduced physical activity in autistic individuals was linked to increased social impairment, as measured by the Social Responsiveness Scale. Long inactivity periods and lower physical activity levels were associated with autism, correlating with less sleep and later sleep onset. Interventional studies are needed to explore if improving sleep and physical activity can improve the quality of life for autistic individuals.

**Lay Abstract:**

Autistic individuals frequently report problems with their sleep, though what aspects of sleep are most affected is not well understood. In this study, we recruited 318 adult autistic participants without intellectual disability and 130 of their non-autistic family members to measure their sleep, physical activity, and daily routines. Study participants wore accelerometer-based wrist-worn devices over 3 consecutive weeks to record their movement and activity. In total, 154 distinct physical activity, sleep, and behavioral traits were identified from the recordings, 52 of which were found to associate with autism. Many of these traits were related to physical activity, where autistic individuals were more likely to be less active for longer periods and have lower overall physical activity levels. Long periods of inactivity also associated with less sleep, with a stronger association in those with autism. For example, for every hour of inactivity, autistic participants had on average ~23 min less of sleep compared to ~17 min in their family members. Autistic individuals with lower levels of physical activity showed higher social impairment as measured by the Social Responsiveness Scale. Overall, lower physical activity may impair sleep and worsen the core features of autism. Interventional studies aimed to increase physical activity may improve the quality of life of autistic individuals.

## Introduction

Autism spectrum conditions (hereafter referred to as autism) are childhood-onset neurodevelopmental disturbances characterized by impairment of social communication and restricted repetitive or stereotyped patterns of behavior (American Psychiatric Association, 2013). Along with these core defining symptoms of autism, individuals often exhibit a range of other associated conditions, including intellectual disability (ID), epilepsy, gastrointestinal problems, motor difficulties, and sleep disturbances. In addition, common co-occurring mental health conditions such as anxiety, depression, and attention deficit hyperactivity disorder (ADHD) are frequently observed (Chaidez et al., 2014; C. R. Johnson et al., 2024; K. P. Johnson & Zarrinnegar, 2024; Reinders et al., 2019).

Sleep disturbances are common in autistic individuals, and reduced sleep duration may serve as an early sign of autism (Humphreys et al., 2014; Missig et al., 2020). Parental or caregiver reports indicate a higher prevalence of disturbed sleep in autistic children (44%–83%) compared to age-matched controls (25%–40%) (Mindell & Meltzer, 2008; Reynolds & Malow, 2011; Veatch et al., 2015). Sleep problems were also frequently reported (39.4%) as co-occurring medical and psychiatric conditions in autistic individuals within the Simons Powering Autism Research (SPARK) cohort (Fombonne et al., 2020). In addition, a questionnaire-based study of children with Asperger syndrome demonstrated an almost twofold increase in the prevalence of short sleep duration and a more than fivefold higher prevalence of sleep onset problems compared to age-matched controls (Paavonen et al., 2008). Autistic individuals often experience a wide range of sleep-related difficulties that persist into adulthood (Jovevska et al., 2020), including problems falling asleep or delayed sleep onset, night awakenings, insomnia, parasomnia, sleep-disordered breathing, persistent daytime sleepiness, and reduced “restorative value of sleep” (Mazurek et al., 2019; Reynolds & Malow, 2011).

Polysomnography (PSG) is an objective measure of sleep architecture and is considered the “gold standard” in the assessment of sleep problems. PSG studies of sleep in both autistic children and adults have corroborated findings from questionnaire-based assessments, such as shorter total sleep time, longer sleep onset latency, and lower sleep efficiency (Diaz-Roman et al., 2018; Elrod & Hood, 2015; Petruzzelli et al., 2021). Although previously reported PSG studies have provided valuable insights into sleep architecture, there are often contrasting reports on differences in sleep stages between autistic individuals and a neurotypical comparison group, such as the percentage of REM sleep (Arazi et al., 2020; Buckley et al., 2010; Kawai et al., 2023). Moreover, an analysis of sleep in autistic children using a parallel PSG and accelerometer-based monitoring, along with parent reports, showed that accelerometer-based monitoring provides an objective, non-intrusive, valid, and lower-cost method for assessing sleep in home settings, alleviating a child’s resistance to a novel environment (Yavuz-Kodat et al., 2019).

Wrist-worn accelerometers are increasingly being used for the assessment of physical activity, sleep, and circadian behavior. Namely, a refined analysis of active and inactive periods can generate indicators of sleep behavior, including, but not limited to sleep duration, daytime inactivity, sleep efficiency, and circadian rhythm patterns (Jones et al., 2019; Roenneberg et al., 2015, 2022). In addition to sensing limb movements (actimetry), these devices also provide data on environmental factors, such as light intensity and near-body temperature. Actimetry studies have been used to monitor sleep in autistic children and adults, offering an objective way to assess sleep and physical activity over several days in familiar home settings (Malow et al., 2016). These studies validated overall differences in sleep and physical activity measures between autistic children and adults relative to controls (Baker & Richdale, 2015; Goldman et al., 2017; Souders et al., 2009). For example, research on autistic individuals and ID has revealed poor sleep efficiency, prolonged sleep latency, and increased number and length of night awakenings (Ballester et al., 2019), further reinforcing the association between autism symptoms and quality of sleep (Elkhatib Smidt et al., 2022; Schreck et al., 2004). Moreover, using a linear multivariate regression analysis, we showed that in adults, increased core autism spectrum traits and executive dysfunction were associated with disruption of several sleep–wake parameters, particularly related to the daily sleep–wake rhythm, and that executive dysfunction was associated with disrupted sleep quality and level of physical activity (Elkhatib Smidt et al., 2022).

In studies of non-clinical populations, individuals exhibiting lower levels of daytime activity tend to experience poorer sleep quality, while those with moderate-to-high levels of daytime physical activity report fewer sleep-related issues, as measured by the Pittsburgh Sleep Quality Index (PSQI) (Arbinaga et al., 2019; Buysse et al., 1989; Memon et al., 2021). This association between activity and sleep quality has also been observed in autism, although the directionality of this relationship is unclear (Benson et al., 2019; Lang et al., 2013; Tse et al., 2019). However, research focusing on activity levels in autism has primarily focused on children, revealing decreased activity levels (MacDonald et al., 2011). Notably, interventions designed to increase physical activity among autistic individuals have shown promise in enhancing executive function and social functioning in meta-analyses (Healy et al., 2018; Liang et al., 2024; Rivera et al., 2025). This suggests that further exploration of sleep and physical activity patterns in autism could offer valuable insights, potentially informing targeted interventions to improve core symptoms.

To evaluate sleep, circadian behavior, and physical activity in autism, we collected accelerometer-based data and autism-related quantitative traits on autistic individuals and a subset of their family members. To reduce phenotypic heterogeneity, we focused our study on the examination of adults (ages 18 to 60) and autistic participants without ID. Moreover, by focusing on participants without ID, we expect to reduce the impact of excessive repetitive movements, which are more common in individuals with co-occurring ID. Using three different algorithms to process accelerometer-derived data, we aimed to characterize sleep, physical activity, and circadian behavior disturbances in autistic adults without ID. Through machine learning methods and linear mixed effects regressions, we identified a wide range of sleep and physical activity features and examined their relationship with core autism traits. Finally, genomic data for the SPARK cohort were used to illustrate a need for future molecular profiling of autistic individuals to resolve phenotypic heterogeneity and better understand molecular mechanisms underlying sleep and activity traits.

## Methods and materials

### Study participants

We recruited participants through the Autism Spectrum Program of Excellence (ASPE) at the University of Pennsylvania (Philadelphia, Pennsylvania) (Elkhatib Smidt et al., 2022; Taylor et al., 2021, 2022) and autistic participants from the Simons Powering Autism Research (SPARK) collection (The SPARK Consortium, 2018; Fombonne et al., 2020). As our study focused on the analysis of autism without ID, we excluded individuals with ID (full-scale intelligence quotient less than 70, as estimated by the Shipley-2 (Shipley et al., 2009)). While co-occurring psychiatric diagnoses *per se* were not an exclusion criterion, we excluded participants with: (1) a history of intellectual disability, (2) severe mood or psychotic symptoms during the last 4 weeks, (3) recent severe aggressive or self-injurious behaviors, and (4) a history of major neurological disorder (e.g., dementia, severe head trauma, and recent seizures). Each ASPE participant completed a 1 h phone interview to collect general demographic information and assess psychiatric and developmental history, social behavior, and autism traits to determine if they met the criteria for autism as defined in the Diagnostic and Statistical Manual of Mental Disorders, Fifth Edition (DSM-5) (American Psychiatric Association, 2013). This assessment identified probands and their family members who met the criteria for autism according to a DSM-5 diagnostic checklist or other psychiatric disorders, as well as family members who did not meet criteria for such disorders (Taylor et al., 2021). This study was approved by the Institutional Review Board of the University of Pennsylvania.

### Phenotypic assessments

All probands (autistic participants) and their potentially non-autistic relatives were assessed for autism-related quantitative traits using self- and informant-reports using the Social Responsiveness Scale (SRS) (Chan et al., 2017; Constantino et al., 2003) and the Broad Autism Phenotype Questionnaire (BAPQ) (Hurley et al., 2007). The SRS scale is used to quantify social communication, social awareness, motivation, and social cognition (Chan et al., 2017). Quantifiable values for pragmatism, rigid thinking, and aloofness were obtained using the BAPQ scale (Hurley et al., 2007). In the final analyses, we used only the informant-reported scores.

Participants wore a GENEActiv™ accelerometer (Activinsights, Kimbolton, United Kingdom) for 21 days on their non-dominant wrist to measure body movements and light exposure using a tri-axial accelerometer. For downstream analysis (see below), up to 21 days of actimetry data for each participant were sequentially numbered (starting at day 1 for the first day of recording) and split into two datasets—even and odd days, with no significant difference in the number of weekend and weekdays across the two datasets (Supplementary Figure 1 A). Activity data, sampled at a frequency of 30 Hz, was processed using three algorithms: GGIR (Migueles et al., 2019), Accelerometer (Doherty et al., 2017; Willetts et al., 2018), and ChronoSapiens (Roenneberg et al., 2015). In the open-source *R*-package GGIR (version 3.1-5), a program designed to process multi-day accelerometer data for sleep and physical activity research, was used to derive day-to-day sleep and activity measures (Migueles et al., 2019). The sleep regularity index (SRI) was calculated using GGIR (Part 4) outputs, providing a quantification of the probability that a person is in the same state (asleep or awake) at the same clock time on consecutive days, ranging from 0 (highly irregular) to 100 (highly regular). The analysis of the actimetry data through GGIR uses an algorithm to detect times of non-wear and to evaluate a range of sleep and physical activity traits for each day of data. We followed standard GGIR quality control methods (Migueles et al., 2019). In the final analysis, we excluded participants with (a) less than 5 days of actimetry data; (b) less than 20 h of data per day; or (c) on average 3 or less hours of sleep per day. For each of the 109 actimetry-derived features, we obtained the within-individual, across-days means, medians, and standard deviations, yielding a total of 218 GGIR variables (all variables listed in Supplementary Table I).

In addition to sleep-related variables, the GGIR program classifies physical activity levels as “inactivity,” “light,” “moderate,” or “vigorous activity” using cutoffs for acceleration values (Migueles et al., 2019). For these classes of physical activity, GGIR also measures time periods in bouts and in blocks. A “bout” is defined as a period during which 80% or more of the time is spent at a specific activity level. For example, inactivity bouts of 30 min or more means that the individual needs to spend 80% of 30 min or more inactive during that period to count as a bout. In contrast, a “block” refers to a continuous segment of a specific activity level lasting a certain amount of time. For example, blocks of inactivity of 30 min or more are periods of continuous inactivity of at least 30 min.

We also analyzed the activity data using ChronoSapiens (CS) (Loock et al., 2021; Roenneberg et al., 2015), a program developed for the analysis of circadian and sleep traits. Activity records were analyzed at a standard 10-min resolution, with non-wear periods identified by detecting continuous stretches of non-activity (zero) lasting more than 100 min. Non-wear was listed as missing data (Loock et al., 2021). The parameters for the models were fit to the activity and computed using a “centered moving” average using a two-harmonic cosine function over the entire time, which is used to determine potential sleep–wake times (Roenneberg et al., 2015). During these potential sleep times, longer periods of inactivity were not classified as non-wear. The daily parameters of the activity rhythm were again calculated by a two-harmonic fit. This analysis from CS yielded 33 variables (Supplementary Table I); thus, the resulting means/medians and standard deviations gave rise to 66 CS variables for each participant.

Finally, we analyzed the same activity data used in CS and GGIR with Accelerometer, a program specifically developed to obtain refined physical activity measures (Doherty et al., 2017; Willetts et al., 2018, 2022). The means and standard deviations for 12 Accelerometer-derived features were included in our analysis, yielding an additional set of 24 variables. All actimetry-derived characteristics from GGIR and Accelerometer were then converted to z-scores using the whole sample as the distribution.

In addition to the ASPE participants (ages 18–60), we performed accelerometer-based evaluation of 173 autistic participants (in the same age range) recruited through the SPARK Research Match (The SPARK Consortium, 2018; Fombonne et al., 2022). We also included data from the caregivers reports in the medical questionnaire for SPARK participants, allowing us to separately analyze those with reported sleep problems or a diagnosed sleep disorder from participants without reported sleep problems. In addition to the actimetry, participants kept a sleep diary that recorded their quality of sleep and other sleep traits each day. To evaluate agreement between these self-reported sleep traits and the actimetry-derived features that match these, we utilized Bland–Altman plots to compute the paired differences between the measures (Bland & Altman, 1999; Marmis et al., 2024).

### Machine learning using elastic net

To assess associations between actimetry-derived sleep and physical activity features (extracted from GGIR, Accelerometer, and ChronoSapiens algorithms) with autism status, we used the elastic net method of machine learning (Friedman et al., 2010; Zou, 2005). This method is particularly robust at selecting features even in the presence of correlated variables. By systematically adjusting a penalization term, the elastic net logistic regression algorithm enabled us to explore a wide range of regression models while effectively eliminating irrelevant variables, reducing their model contributions to zero. To improve the algorithm, the hyperparameter α was tested to find the ideal value between 0 and 1, referencing the fraction of the L1 regularization to L2 regularization used in the analysis. Briefly, we can tune the ML analysis to better refine and improve our models by finding the best value for this term. To achieve this, 1000 iterations of the machine learning were performed at values of α ranging from 0 to 1 in increments of 0.05. The best-performing α cutoff was determined as the cutoff with the highest median area under the curve (AUC) (Supplementary Figure 2).

We employed complementary approaches for model validation. As described, data for each participant were sequentially split into even and odd datasets. We compared a distribution of week and weekend days data from odd days, which were used to train and validate the model, utilizing a 10-fold cross-validation approach. Briefly, participants were randomly split into 10 groups, and the model was trained on the data from 9 of the groups and then validated on the data from the last, independent 10th group. Data from even days served as a separate quasi-independent testing set to control for potential overfitting of the models. The mean and standard deviation for each actimetry-derived sleep and physical activity trait were calculated separately within the odd day and even day datasets to evaluate whether the overall average and intra-individual variance of a trait differed in the two datasets. As autism was a binary outcome variable, model error was determined and compared using binomial deviance in the elastic net logistic regression models.

We also performed the elastic net analysis using the medians and standard deviations of the actimetry-derived features, rather than the means and standard deviations. We then compared these median/SD models with the mean/SD models for similarities, by identifying how many variables were selected in both algorithms with the same direction of effect.

To identify highly associated variables, we performed hierarchical clustering on the correlation matrix using the corrplot function in the corrplot package (Taiyun Wei, 2024). Within the identified clusters, the variable with the strongest association with autism was highlighted as the “lead” variable for the cluster.

In addition to the method described above, we implemented a more conventional machine learning approach to further validate our findings. Forty-one participants (~15%) were randomly selected and removed from the model selection methodology and training steps to be a fully independent validation set. For the remaining participants, we calculated the best-fit model using 10-fold cross-validation. The best-fit model was then tested in this fully independent sample of 41 participants.

### Association between physical activity and sleep characteristics in the context of autism

Mixed effects linear regression was used to investigate how autism status may modulate the relationship between sleep and physical activity traits. In these regressions, we focused on the 11 sleep variables: sleep onset time, wake-up time, sleep period duration, sleep duration in sleep period, wake after sleep onset (WASO), number of awakenings, duration of sleep, number of awakenings of at least 5 min, sleep efficiency, and total sleep time. In contrast to the mean and standard deviation for each variable used in the elastic net algorithm, we analyzed each 24 h day/night cycle for every participant to evaluate the relationship between the level of daytime physical activity and the following night’s sleep. A linear mixed effects model was created for each trait combination using the lme4 R package (Bates et al., 2015), with the following covariates: age, age^2^, sex, autism status, the physical activity trait of interest, the interaction between autism and the physical activity traits, and two random effect variables to account for repeated measures within individuals (e.g. multiple days) and potential family effects, by including a random intercept for the individual and for the family. To account for non-linear relationships between age and sleep and physical activity characteristics, we included the age^2^ variable in the analysis. The significance for the physical activity variable and autism diagnosis were calculated in a model excluding the interaction term, while the interaction significance was evaluated by including the interaction between physical activity and autism diagnosis. To further ensure our models are not overfitting, we conducted likelihood ratio tests (LRTs) to evaluate the main effects of the physical activity features and to determine whether the addition of the interaction term (autism-by-physical activity) improved the model’s performance significantly. This was performed using the *anova* function in R comparing different linear mixed regression models with and without the physical activity trait, and with or without the interaction term.

### Identifying actimetry-derived features associated with autism trait questionnaire results

To identify which actimetry-derived variables were selected from the elastic net algorithm associated with questionnaire-derived autism traits, we utilized linear stepwise regression analyses. Using this method, we selected features that demonstrated strong associations with traits measured by the SRS(Chan et al., 2017) and the Broad Autism Phenotype Questionnaire (BAPQ) (Hurley et al., 2007). A forward stepwise regression was performed on the age-adjusted total informant-report scores for the SRS and BAPQ, with the Akaike information criterion (AIC) guiding the inclusion or removal of variables at each step of the model.

### Validation of findings in a set of SPARK participants

To further evaluate the association of the identified actimetry-derived features from the elastic net models, we evaluated how these traits differed in the autistic participants from the SPARK consortium (The SPARK Consortium, 2018; Fombonne et al., 2022). We used the same 3-week protocol for data collection and quality control that was used for the ASPE participants. We leveraged caregiver reports in the SPARK medical questionnaire (Consortium, 2018; Fombonne et al., 2020) and separated SPARK participants based on the presence (SPARK SP) or absence (SPARK noSP) of reported sleep problems. They were then compared to the unaffected relatives (UR) and the autistic probands (AP) in the ASPE cohort. *P*-values were converted to false discovery rate (FDR) values using the Benjamini and Hochberg procedure (Benjamini et al., 2001; Benjamini & Hochberg, 1995).

### Preliminary genetic analysis of sleep traits in autistic and non-autistic family members

We analyzed the SPARK Integrated Whole Exome Sequencing (iWES-v3) dataset, generated using 150 bp paired-end reads on Illumina NovaSeq 6000 machines available through the SFARI Base (https://base.sfari.org/). Variants were called with both DeepVariant (v1.4.0) and GATK (v4.2.6.1), and only variants detected by both callers were included in the analysis. Using mixed effects logistic regression, we assessed whether individuals with reported sleep problems (Fombonne et al., 2020) carry a higher burden of rare (MAF ⩽ 0.1%) and deleterious (CADD ⩾ 20 and/or REVEL ⩾ 0.5) variants in 133 genes manually curated by detailed literature review (referred to in this article as “sleep genes”) (Lee et al., 2023). In the analysis, we accounted for family structure and covariates including age, sex, autism diagnosis, and race. In addition, actigraphy-derived sleep traits selected by the elastic net machine learning approach were compared across autistic participants, their non-autistic relatives, and SPARK individuals, stratified by autism and variant carrier status, with significance tested by Wilcoxon rank-sum and corrected for multiple comparisons.

## Results

### Study overview and demographic data

To determine sleep, activity, or circadian traits potentially associated with autism in adults without ID, we leveraged accelerometer-based data collected over 8249 days from autistic individuals and their family members recruited through the ASPE program and autistic individuals from the SPARK Collection. Demographic data for 145 autistic participants in the ASPE cohort and their 130 non-autistic relatives (first- and second-degree) and 173 autistic participants in SPARK are listed in Table 1. In our cohorts, family members of ASPE probands were on average older than both the autistic participants in the ASPE and SPARK groups (p < 0.05), with more females (57% vs 43%, chi-square test, p < 0.05), and reported fewer diagnoses of co-occurring conditions, such as depression, ADHD, and anxiety compared to the autism groups (p < 0.05), with no difference in socioeconomic status (SES) derived from median household income by zip code between these study groups (Mann–Whitney U test, Table 1).

**Table 1.** Demographic data for all participants.

	Autistic ASPE participants (n = 145)	Non-autistic ASPE family members (n = 130)	Autistic SPARK participants (n = 173)
Age, mean (sd)	35.4 (12.4)	42.0 (12.4)	32.3 (9.0)
Sex, female (%)	64 (44.1%)	76 (58.5%)	61 (35.3%)
BAPQ z-score scaled, mean (sd)	0.714 (0.82)	−0.59 (0.56)	–
SRS z-score scaled, mean (sd)	0.58 (0.89)	−0.55 (0.59)	–
Participants with depression (%)	68 (46.9%)	42 (32.3%)	98 (56.6%)
Participants with anxiety (%)	81 (55.9%)	38 (29.2%)	109 (63.0%)
Participants with ADHD (%)	65 (44.8%)	21 (16.2%)	68 (39.3%)
Participants with Reported Sleep Problems (%)	–	–	43 (24.8%)
Caucasian (%)	99 (68.3%)	102 (78.5%)	153 (88.4%)
Black (%)	9 (6.2%)	9 (6.9%)	7 (4.0%)
Asian (%)	5 (3.4%)	6 (4.6%)	7 (4.0%)
Native American (%)	7 (4.8%)	6 (4.6%)	12 (6.9%)
Other (%)	5 (3.4%)	2 (1.5%)	6 (3.5%)
No Selection Made (%)	20 (13.8%)	5 (3.8%)	6 (5.2%)
Median Household Income of Zip Code, mean (sd)	$92,168 ($36,872)	$101,000 ($33,857)	-
Employed (%)	88 (60.7%)	99 (76.2%)	98 (56.6%)
Unemployed (%)	45 (31.0%)	22 (16.9%)	58 (33.5%)

**Figure 1.**
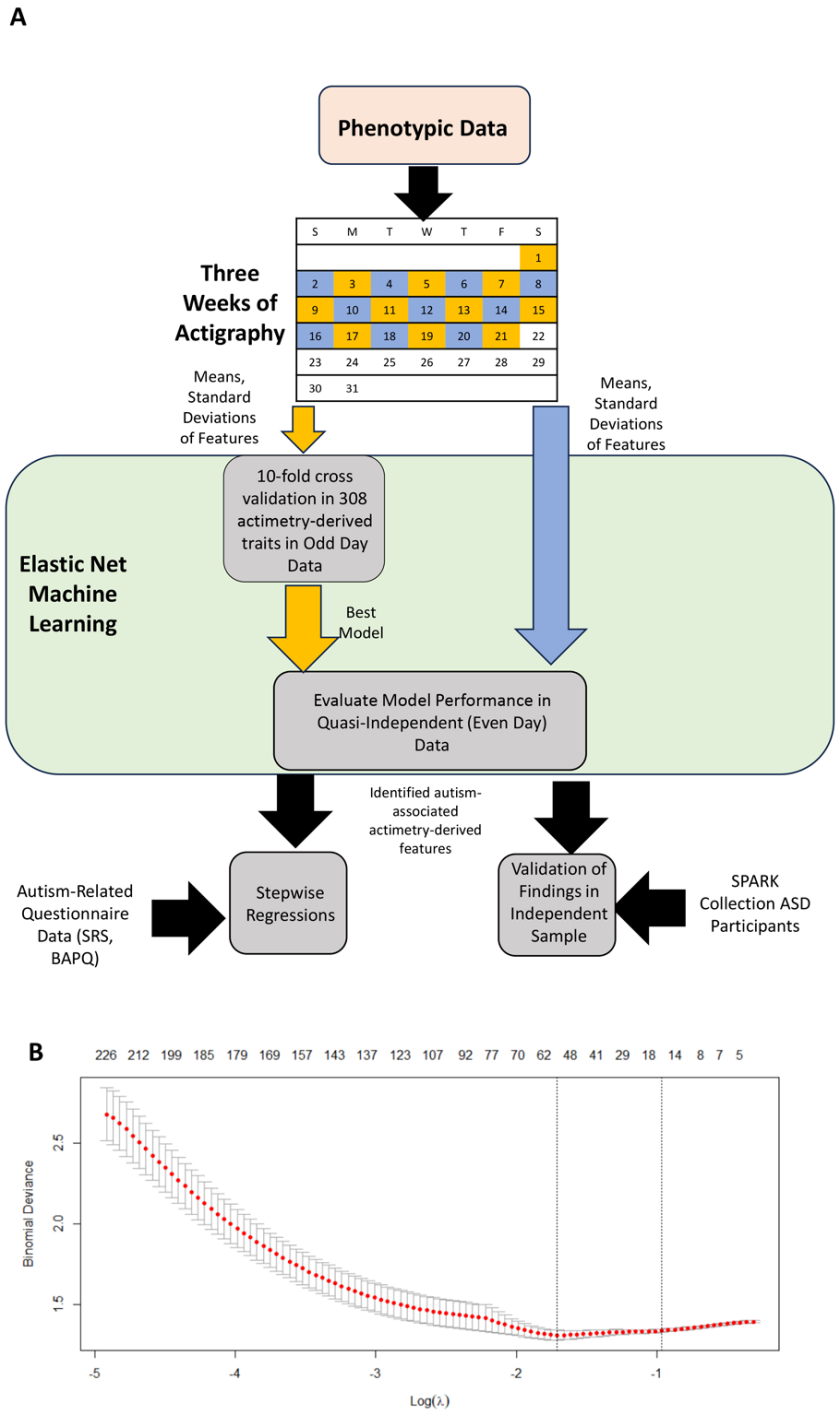
Prioritization of accelerometer-derived sleep and activity traits using machine learning. (a) ASPE study participants were recruited (145 autistic individuals and 130 of their family members without autism); their autism-associated behavioral traits were assessed using questionnaires, and their sleep and activity patterns were derived from 3 weeks of accelerometer-based data. The accelerometer-based data were split into two sets; even (blue) and odd (yellow) days, where the odd days were used for elastic net to build a prediction model for autism. The best-performing model was then evaluated in the even days dataset. (b) Plot showing model selection in elastic net using fivefold cross-validation. Models were evaluated using binomial deviance. The top numbers refer to the number of variables within the model.

The overall research strategy is shown in Figure 1(a). On average, we analyzed 18.2 days per participant, with a mean (SD) of 23.6 (0.78) h of data per day after excluding those with fewer than 5 days of data, or days with less than 20 h of recordings. Self-reported total sleep time, sleep offset, and sleep onset showed fair to good correlations (*r* = 0.44, 0.68, 0.73, respectively) when compared to corresponding GGIR-derived characteristics (Migueles et al., 2019) based on reported guidelines for evaluating correlations (Cicchetti & Sparrow, 1981). In contrast, sleep efficiency was poorly correlated between self-reported and actimetry-derived data (*r* = 0.038). This finding aligns with other studies, where participant self-reports often do not match actimetry-derived or PSG data (Landry et al., 2015; Zinkhan et al., 2014).

Bland–Altman analyses of sleep diaries and GGIR-derived sleep measures revealed systematic biases and substantial variability in agreement between these self-reports and objective measures (Supplementary Figure 3). For wakeup time, the mean bias was minimal (approximately 0 min), indicating good average agreement, though individual differences ranged widely within the 95% limits of agreement (LoA). Sleep onset showed a slight systematic bias with diary reports occurring earlier than GGIR estimates on average, with considerable individual variability evident in the 95% LoA. Total sleep time demonstrated similarly minimal average bias but with substantial individual-level disagreement, as reflected in the wide 95% LoA spanning approximately ± 200 min. Sleep efficiency showed the poorest agreement, with diary reports systematically overestimating efficiency compared to GGIR by approximately 10–15 percentage points on average, and 95% LoA extending from approximately −25 to +50 percentage points, indicating substantial measurement disagreement at the individual level.

### Prioritization of sleep and physical activity features using machine learning

To investigate which actimetry-derived features are most strongly associated with autism in an unbiased, data-driven approach, we utilized the elastic net machine learning method on the mean and SD of 154 (308 total) actimetry-derived circadian, sleep, and physical activity variables (Supplementary Table I; correlations in Supplementary Figure 4). In our best-fitting model (AUC 0.812 and 0.756, odd and even days, respectively; Table 2), 52 variables were selected as associated with autism diagnosis (Supplementary Table II). In the top 10, both the average amount of sedentary time and the variability in sedentary time were associated with autism, with autistic individuals having more time spent sedentary and more consistent sedentary time (lower variability) between days. In addition, the average time spent in mixed physical activity was significantly lower in autistic individuals compared to individuals without autism. Variance in the duration of the sleep period also showed association with autism status; that is, autistic individuals had more within-person variability in their sleep duration than those without autism.

**Table 2.** Elastic net model performance as measured by the area under the curve (AUC).

	Odd Day AUC	Even Day AUC
GGIR Only	0.784 (0.730–0.837)	0.747 (0.689–0.805)
Accel Only	0.731 (0.672–0.790)	0.691 (0.628–0.753)
ChronoSapiens Only	0.732 (0.673–0.791)	0.706 (0.644–0.768)
GGIR, Accel, and ChronoSapiens	0.805 (0.753–0.857)	0.751 (0.694–0.808)
Only Age and Sex	0.664 (0.600–0.728)	
GGIR, Accel, and ChronoSapiens (with Age and Sex)	0.812 (0.761–0.862)	0.756 (0.700–0.813)
Means Only	0.748 (0.691–0.806)	0.738 (0.680–0.797)
Variances Only	0.781 (0.726–0.836)	0.704 (0.643–0.765)

Model performance is shown in both the odd-day and the even-day datasets.

Out of the elastic net-selected actimetry-derived traits, eight individual traits showed associations with autism in both their means and the variances (Table 3). These included physical activity features such as time spent sedentary and time spent in mixed physical activity, and circadian variables such as onset time for 10 h of highest activity period (M10). Of the 52 total variables selected, 27 (51.9%) were the standard deviations of the traits.

**Table 3.** Actimetry-derived features where both the mean and the standard deviations were selected by the elastic net.

Trait	Mean Beta	Variance Beta
Duration of unbouted light physical activity	−0.023	−0.034
M10 average peak light	−0.0021	−0.0083
M10 onset time	0.0020	−0.0062
Mixed physical activity duration	−0.093	−0.014
Number of blocks per day of unbouted light physical activity	−0.026	−0.036
Number of blocks per day of unbouted moderate physical activity	−0.0087	−0.037
Time spent sedentary	0.070	−0.088
Weartime	−0.043	0.047

The analysis was also conducted using the medians and standard deviations rather than the means and standard deviations, to potentially account for data that is not normally distributed. The AUC for this full model was 0.817 and 0.752 for odd and even days, respectively, therefore not statistically significantly different from the means and standard deviations model (0.812, 0.756). The median/SD model identified 47 actimetry-derived variables associated with autism, with 40 of these variables (85%) overlapping with the mean/SD model.

To further strengthen our findings, we created models using elastic net following a more traditional approach: using a fully independent sample, where 41 participants were removed from the model selection and calculation steps, and were used as a fully independent sample for model validation. We observed lower, but still significant AUC values (0.727, 0.693) for this model compared to the even/day split models. This may be due to the lower sample sizes for model creation and/or through more rigorous control for overfitting. Of the 46 actimetry-derived variables selected in this model, 35 (76%) are shared with the mean/SD model described above from the odd/even day split data suggesting strong variable overlap (Supplementary Tables III and IV).

**Figure 2.**
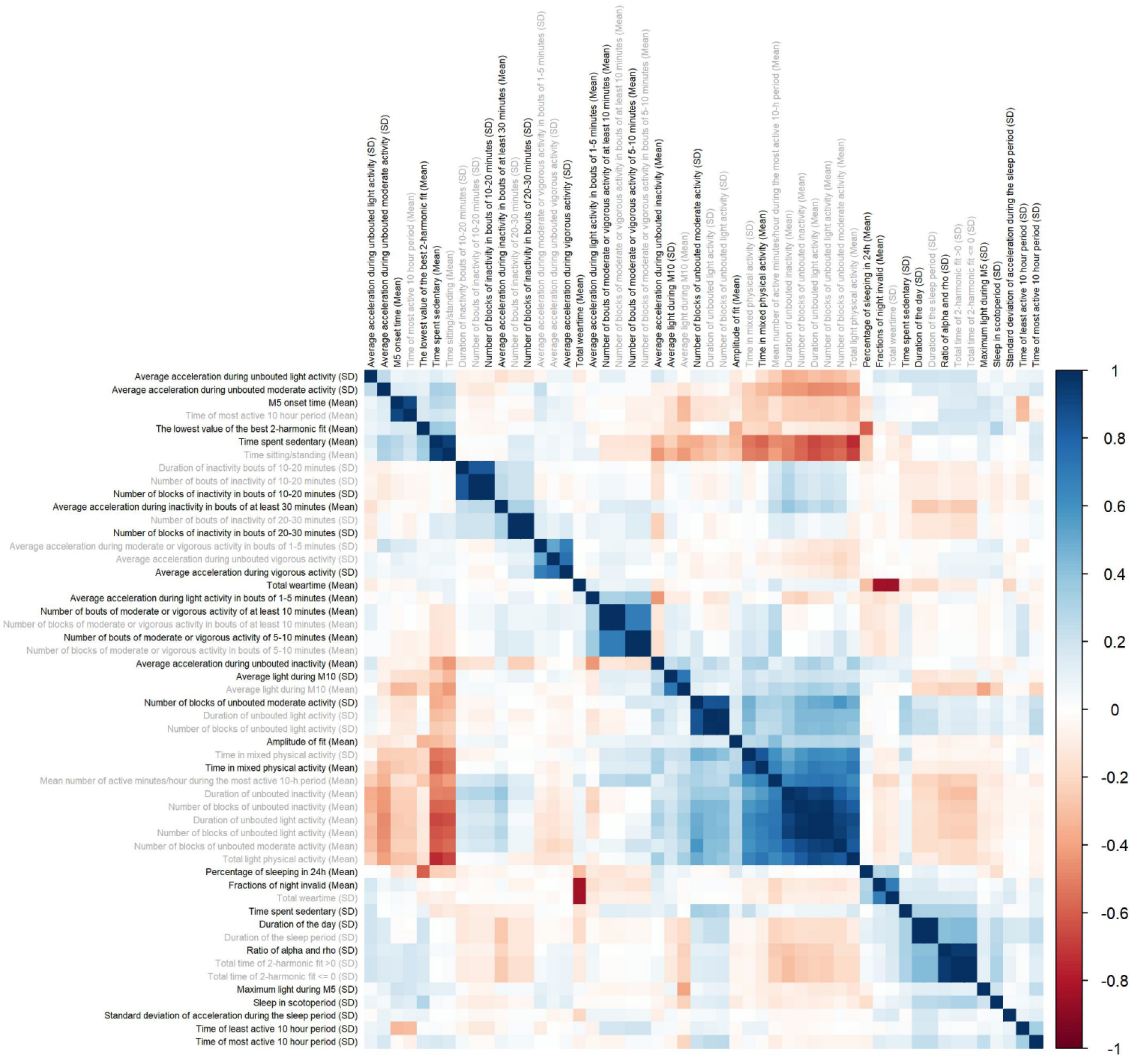
Relationships between 52 actimetry-derived features selected as associated with autism by elastic net. Pearson correlations between the 52 actimetry-derived traits identified through the elastic net regression. Hierarchical clustering identified 28 highly correlated clusters. In each of these clusters, we identified the trait with the strongest association with autism within the cluster (bold) while other variables in the clusters are marked in gray.

To reduce the redundancy of variables selected by elastic net, we performed a correlation cluster analysis on the selected actimetry-derived traits (Figure 2). The hierarchical clustering revealed 28 clusters of actimetry-derived features. This step identified 28 traits with the strongest association with autism, representing each cluster (Table 4).

**Table 4.** The top 28/52 actimetry-derived traits associated with autism selected by elastic net.

Name	Beta	Algorithm
Maximum light during M5 (SD)	0.09451	GGIR
Time in mixed physical activity (mean)	−0.09328	Accelerometer
Time spent sedentary (SD)	−0.08824	Accelerometer
The lowest value of the best 2-harmonic fit (mean)	0.07197	ChronoSapiens
Time spent sedentary (mean)	0.07009	Accelerometer
Duration of the day (SD)	0.06139	GGIR
Average acceleration during unbouted moderate activity (SD)	0.05560	GGIR
Range of oscillation (mean)	−0.04913	ChronoSapiens
M5 onset time (mean)	0.04840	GGIR
Time of least active 10-h period (SD)	0.04682	GGIR
Average acceleration during inactivity in bouts of at least 30 min (mean)	0.04566	GGIR
Total weartime (mean)	−0.04336	Accelerometer
Number of blocks of unbouted moderate activity (SD)	−0.03684	GGIR
Average acceleration during vigorous activity (SD)	0.03511	GGIR
Fractions of night invalid (mean)	0.03486	GGIR
Ratio of alpha and rho (SD)	0.02891	ChronoSapiens
Number of blocks of inactivity in bouts of 10–20 min (SD)	0.02467	GGIR
Average acceleration during unbouted light activity (SD)	0.02400	GGIR
Standard deviation of acceleration during the sleep period (SD)	0.02242	GGIR
Percentage of sleeping in 24 h (mean)	−0.02240	ChronoSapiens
Number of bouts of moderate or vigorous activity of 5–10 min (Mean)	0.01719	GGIR
Number of blocks of moderate or vigorous activity in bouts of 5–10 min (mean)	0.01017	GGIR
Average light during M10 (SD)	−0.00829	GGIR
Average acceleration during unbouted inactivity (mean)	−0.00687	GGIR
Time of most active 10 h period (SD)	−0.00623	GGIR
Average acceleration during light activity in bouts of 1–5 min (Mean)	0.00148	GGIR
Sleep in scotoperiod (SD)	0.00117	ChronoSapiens
Number of blocks of inactivity in bouts of 20–30 min (SD)	−0.00005	GGIR

To reduce the number of variables from 52 to 28, one variable was selected from each cluster after performing correlation clustering analysis. We selected one variable in each cluster by identifying the actimetry-derived variable most associated with autism in each cluster.

To further compare the importance of the average values of the actimetry-derived features and their day-to-day variability, we generated separate elastic net models for mean values only and a corresponding model with only the standard deviations. The analysis of mean values yielded AUCs of 0.748 and 0.738 for odd and even days, respectively, while including only the standard deviation produced AUCs of 0.781 and 0.704 (Table 2). This suggests that the day-to-day variation of sleep, circadian, and physical activity traits may be of similar importance to study compared to the average magnitude of that feature, that is, the variation in time spent sedentary may be as associated with autism as the average amount of sedentary time.

Autism commonly co-occurs with other neuro-psychiatric conditions, which may impact the results of our findings. To evaluate how these co-occurring conditions may also be selected by the elastic net analysis, we compared the performance of the autism model with ADHD, anxiety, or depression co-occurring in ASPE participants. We observed a significant performance with ADHD (AUC 0.704, 0.657; odd and even, respectively) but not with anxiety (AUC 0.564, 0.539) or depression (AUC 0.545, 0.522). Due to the high co-occurrence of ADHD, especially within our autism cohort (and within autistic individuals), our model appears predictive of both autism and ADHD status within families of autistic individuals (Supplementary Table V).

### Assessing seasonal confounding in the data

To evaluate whether our findings may be influenced by seasonal variation in data collection, we first investigated the distribution of recording days across seasons between participants with and without autism. We found a significant difference in the seasonal distribution of data, with participants without autism contributing more data during the summer months compared to autistic participants (p < 0.05, chi-square test; Supplemental Figure 1B).

Given this imbalance, we next examined whether any of the machine learning-identified traits varied significantly by season (Supplementary Figure 5). We used mixed effects regression models to test for seasonal associations with each actigraphy-derived trait, accounting for the random effects of multiple observations per participant, potentially across seasons. After adjusting for multiple comparisons using the Benjamini-Hochberg procedure, 3 of 44 autism-associated machine learning traits were significantly associated with season: maximum peak light during M5 (most active 5 h period), average peak light during M10 (most active 10 h period), and sleep duration during the designated sleep period (Supplementary Table VI). Post hoc pairwise comparisons revealed specific seasonal differences for each significant trait. For M5 maximum peak light, significant differences were observed between autumn-summer, autumn-winter, spring-winter, and summer-winter. For M10 mean light, all season pairs except autumn-spring showed significant differences. For sleep duration during the sleep period, significant differences were found between autumn–summer, spring–summer, spring–winter, and summer–winter pairs.

### Relationship between sleep and physical activity in autism

**Figure 3.**
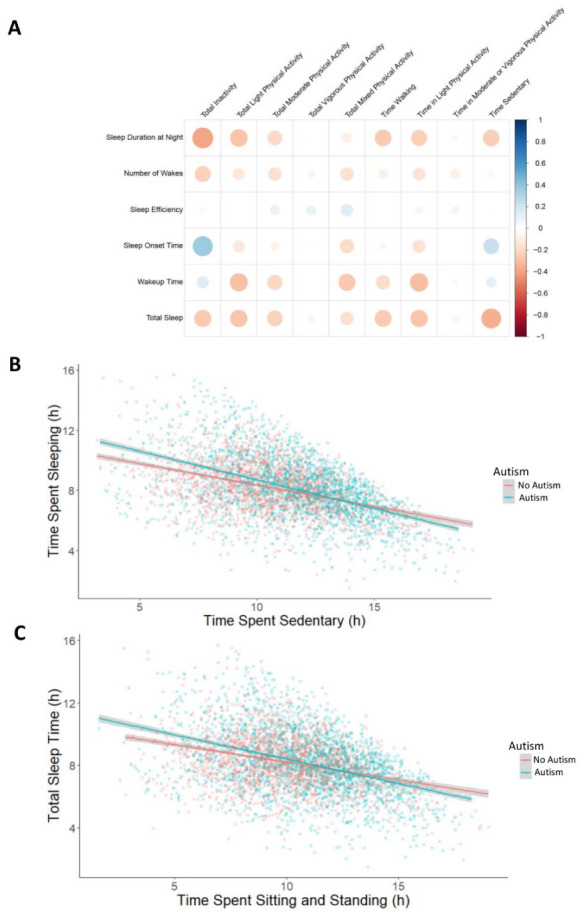
Relationship between physical activity and sleep in autism. (a) Rank Spearman correlation matrix for average physical activity traits compared to average sleep traits. Total inactivity and sedentary behavior correlated negatively with duration of sleep and positively with time of sleep onset. (b) The relationship between total sleep time and time spent sedentary, split by autism status. (c) Relationship between time spent sitting/standing and total sleep time, split by autism status.

A higher level of physical activity has previously been shown to be associated with higher reported sleep quality (Kredlow et al., 2015; Memon et al., 2021). Here, we evaluated how autism status may modify these relationships between physical activity features and total sleep time, number of WASO, sleep onset time, and wakeup time. In a Spearman rank correlation with physical activity and sleep variables, individuals with a higher time spent sedentary had shorter sleep duration and a later sleep onset, regardless of their autism status (Figure 3(a)).

To assess if autism status modulates the observed relationships, we used the 11 core sleep traits from the three algorithms (GGIR, CS, and Accelerometer) as the outcome variables in linear mixed effects regressions (Supplementary Table VII). Briefly, four of the 11 sleep traits had at least one significant interaction between an actimetry-derived non-sleep feature and autism status (Supplementary Table VII), indicating that autism may modulate these phenotypes. For instance, autism status modulated the observed relationship between time spent sedentary and total sleep time, where autistic individuals experienced a larger reduction in total sleep time when they have a longer period spent sedentary than their non-autistic relatives (Figure 3(b)). Specifically, for every 1 h of sedentary time, autistic individuals had, on average, 23.5 min less total sleep time, whereas for every 1 h of sedentary time, non-autistic individuals had only 17.1 min less total sleep time. After controlling for age, age^2^, sex, individual and family differences, autistic individuals had a statistically significant average decrease of 15.6 min of sleep for each hour of sedentary time, while those without autism had a decrease of 13.0 min on average. After accounting for these features, the difference was reduced but still statistically significant. For example, the average time spent sedentary for our autistic participants is 10.98 h, therefore based on our model, autistic individuals seem to sleep 28–71 min less than their non-autistic relatives. As the Accelerometer algorithm differentiates between sedentary behavior and sleep, this cannot be explained by daytime naps. Similarly, longer sitting/standing time also interacted with autism status, with autistic individuals losing more total sleep time with longer sitting/standing time (Figure 3(c)).

### Evaluation of actimetry-derived characteristics associated with autism-related phenotypes

**Figure 4.**
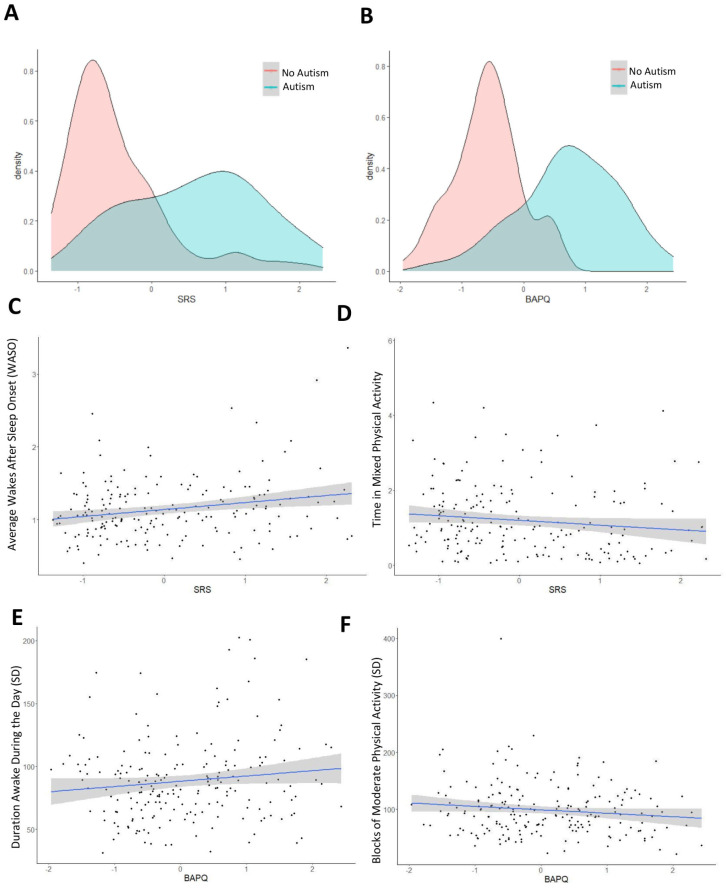
Phenotypic correlation of autism and accelerometer-derived traits: (a) A correlation matrix showing the correlations between autism traits assessed based on two questionnaires: Social Responsiveness Scale (SRS), and (b) the Broad Autism Phenotype Questionnaire (BAPQ). (c) Wakes after sleep onset (WASO) and time in mixed physical activity (d) correlate with the total SRS scores. (e) Duration awake during the day and moderate physical activity (f) correlate with BAPQ.

To further validate the associations identified by the elastic net algorithm, we compared these actimetry-derived variables with quantitative autism-related traits, collected using the SRS (Chan et al., 2017) and the Broad Autism Phenotype Questionnaire (BAPQ) (Hurley et al., 2007) (Figure 4(a) and (b)).

We evaluated how the 28 actimetry-derived features identified through our elastic net approach were associated with quantitative autism-related traits, derived from the SRS and BAPQ (Figure 4(a) and (b)). Higher WASO was associated with a higher social impairment (Figure 4(c)). We employed a bi-directional, stepwise regression to identify significant actimetry-derived traits that were predictive of SRS and BAPQ scores across all participants, including autistic participants and their relatives (Taylor et al., 2021, 2022). The actimetry-derived features were more strongly associated with SRS scores than BAPQ scores, with an *R*^2^ of 0.16 compared to 0.12, and an adjusted *R*^2^ of 0.10 compared to 0.07. The stepwise regressions selected eight actimetry-derived traits associated with the severity of social responsiveness measured by the SRS, of which three were significant (Supplementary Table VIII). For example, lower average levels of mixed physical activity were associated with severe autistic traits (Figure 4(d)). Our method identified five actimetry-derived traits associated with broad autism traits (BAPQ), with two of them significant: higher variation of the duration awake during the day and higher variability of moderate physical activity (Figure 4(e) and (f); Supplementary Table IX).

### Validations of findings in the SPARK collection

**Figure 5.**
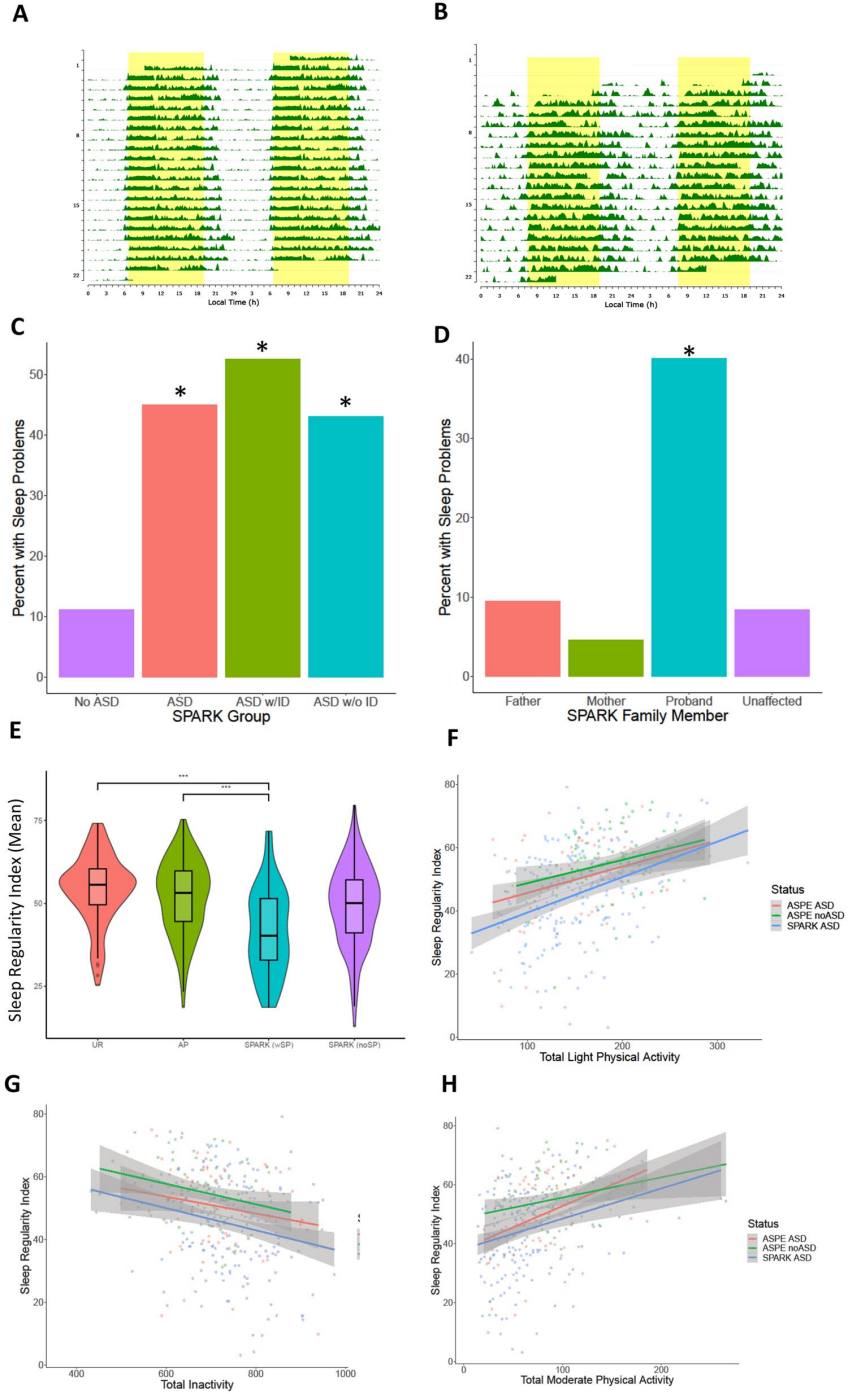
Analysis of sleep regularity and reported sleep problems in the SPARK cohort. Actimetry-derived double plots visualized using the ChronoSapiens (Roenneberg et al., 2015) package. These plots show the activity throughout the three weeks of data collection, where (a) shows a highly regular activity pattern of an individual and (b) shows a highly irregular activity pattern of an individual. (c) Reported sleep problems in the SPARK medical questionnaire (n = 146,843). (d) In European ancestry quartets, where data is available for both parents and an unaffected sibling (n = 6,196; 1549 in each group), the autistic probands are more likely to report sleep problems than any other family member role. (e) The sleep regularity index (SRI) scores between groups showed that the autistic individuals were significantly more irregular in their sleep than the ASPE unaffected relatives. The SPARK individuals with reported sleep problems were also more irregular than the ASPE autistic individuals. UR: ASPE unaffected relatives (n = 130), AP: autistic ASPE participants (n = 145), SPARK (noSP): SPARK autistic probands with no reported sleep problems (n = 135), SPARK (SP): SPARK probands with autism and reported sleep problems (n = 45). (f) Comparisons of the relationship between total light physical activity, time spent inactive (g), and total moderate physical activity (h) and the SRI scores, split between ASPE autism, ASPE without autism, and the SPARK autism participants.

To further validate our findings from the elastic net model, we evaluated the selected actimetry-derived traits in the fully independent sample from the SPARK consortium with representative actigraphy plots (Figure 5(a) and (b)). Based on the responses of their caregivers to the SPARK medical questionnaire (Consortium, 2018; Fombonne et al., 2020), autistic individuals were more likely to report sleep problems than family members without autism (Figure 5(c) and (d)). We compared ASPE and SPARK participants separated into the following groups: (a) ASPE autistic probands, (b) ASPE relatives without autism (UR), (c) SPARK AP with reported sleep problems (SPARK SP), and (d) SPARK autistic participants without reported sleep problems (SPARK noSP). To evaluate the overall regularity of sleep patterns in autism, we calculated the SRI (Fischer et al., 2021; Phillips et al., 2017). Briefly, the algorithm calculates the percentage probability of an individual being in the same state (sleep vs wake) at any two time points 24 h apart (Phillips et al., 2017). A score of 100 means that the person wakes up and goes to sleep at exactly the same time between days, and a score of zero indicates that the individual is asleep or awake at random. Therefore, a higher score denotes more regular sleep/wake patterns, while a lower score shows lower regularity of daily sleep/wake patterns. Autistic individuals, both in ASPE and SPARK, had significantly lower SRI scores than the ASPE relatives with no psychiatric conditions (Figure 5(e)). In addition, SPARK individuals who reported sleep problems had an even lower regularity in their sleep values (Figure 5(e)) compared to autistic individuals whose caregivers did not report sleep problems for that individual. Correlations between the SRI and physical activity showed that higher light activity, lower inactivity, and higher moderate physical activity correlated to more sleep regularity. Autistic individuals in both SPARK and ASPE have overall lower SRI at the same level of physical activity (Figure 5(f) to (h)).

**Figure 6.**
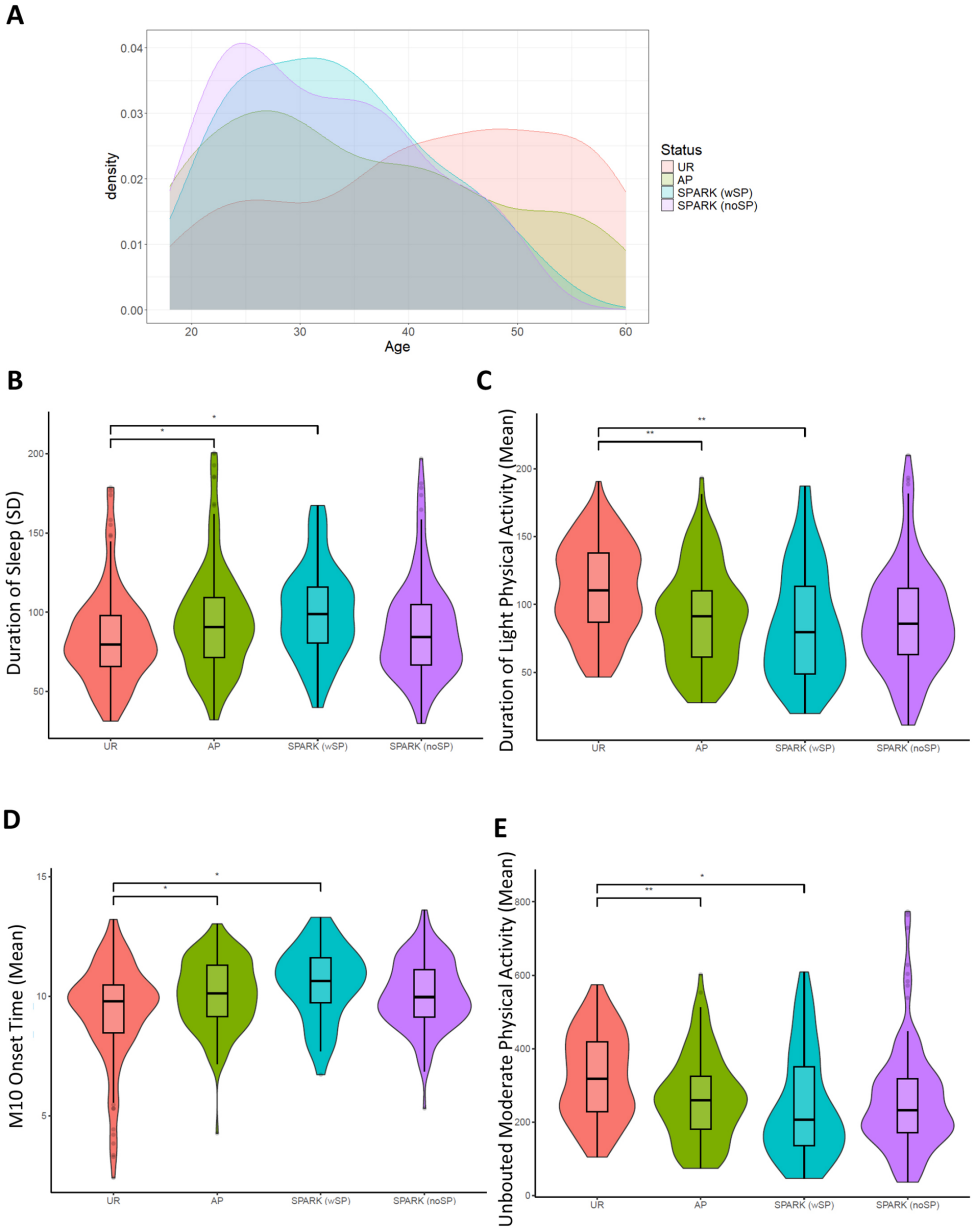
Phenotypic correlation of autism and accelerometer-derived traits: (a) Age distributions of the groups in our study. UR: unaffected relatives in ASPE (n = 130), AP: ASPE autistic probands (n = 145), SPARK (noSP): SPARK autistic probands without reported sleep problems (n = 135), SPARK (SP): SPARK autistic probands with reported sleep problems (n = 45). (b) Variability in duration of sleep between the groups showed that the autistic groups had more variability. (c) Autistic groups had less light physical activity, later M10 onset (d), and less unbouted moderate physical activity (e) than the ASPE relatives without autism. P-values are corrected using the Benjamini-Hochberg procedure, with cutoffs: * = < 0.05; ** = < 0.005; *** = < 0.0005.

Next, we investigated how the 28 actimetry-derived features selected by machine learning may differ between the SPARK groups (Figure 6(a)). Of the 28 actimetry-derived features selected by elastic net, seven were found to be significantly different between the SPARK SP and the UR groups after Benjamini–Hochberg corrections and controlling for age, sex, and age^2^. These included higher variability in the duration of sleep at night (Figure 6(b)), lower duration of light physical activity (Figure 6(c)), later M10 onset time (Figure 6(d)), and less moderate physical activity (Figure 6(e)).

### Genetic investigations of sleep and activity disturbances

**Figure 7.**
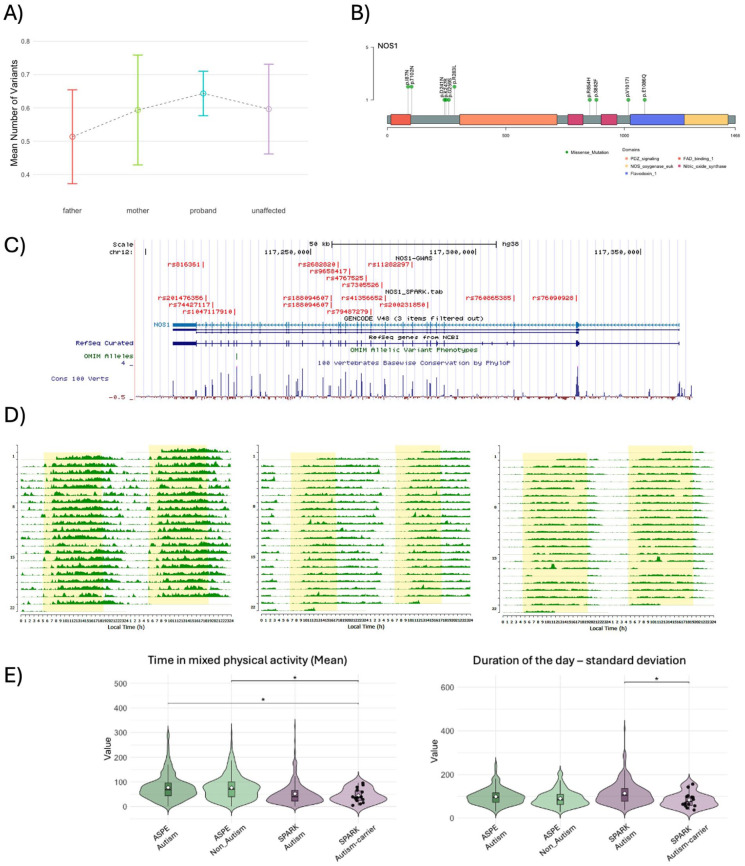
Genetic analysis of rare variants in clock/sleep genes. (a) Burden analysis of rare variants in clock/sleep genes (n = 133) across 1549 SPARK families (parents, autistic individuals, and non-autistic siblings). (b) Lollipop plot of rare variants identified in the NOS1 gene, showing their distribution across protein domains. These variants were found in SPARK autistic subjects with actigraphy. (c) Genome Browser map of the NOS1 locus with chromosomal position of SNVs with significant association with insomnia in a GWAS study (Watanabe et al., 2022) (NOS1-GWAS) and rare (minor allele frequency ⩽ 0.1%), exonic predicted deleterious (CADD ⩾ 20; RAVEL ⩾ 0.5) SNVs (rsIDs) in SPARK participants (NOS1-SPARK (d) Representative actigraphs illustrating sleep–wake patterns for three individuals carrying rare NOS1 variants. (e) Violin plots showing significant differences in variability in day duration and time in mixed physical activity.

The availability of genomic data for the participants in the SPARK cohort offered the opportunity to initiate the investigation of the role of gene variants on the reported sleep disturbances and activity patterns. In this study, we asked if variants in a set of 133 genes selected based on reported circadian and sleep disturbances in studies in model organisms (Lee et al., 2023) contribute to reported sleep problems. We initially performed genetic analysis of Whole Exome Sequence (iWES-v3) for 1,549 SPARK quartet families (presented in Figure 5(d)) and examined the burden of deleterious variants in this gene set across family members with reported sleep problems and/or diagnosed sleep disorders. We did not detect significant differences in the burden of rare, predicted deleterious variants across family members—mothers, fathers, AP, and their unaffected siblings, although we detected a slightly elevated burden in probands (Figure 7(a)). The number of SPARK subjects with genomic and actimetry data was too low to evaluate the impact of rare variants on quantitative sleep and activity traits selected by the elastic net models. However, to illustrate the need for molecular subtyping of individuals, we report genotype-phenotype comparison in the nitric oxide synthase (*NOS1*) gene as an example. Based on previous experimental evidence, *NOS1* knock-out mouse mutants have reduced REM sleep and attenuated homeostatic response to sleep deprivation (Chen et al., 2003; Morairty et al., 2013). Moreover, six single-nucleotide variants at the *NOS1* locus were reported as significant in the insomnia Genome Wide Association Study (Watanabe et al., 2022) (Figure 7(c)). Among SPARK subjects with actimetry data, we identified 13 individuals with rare, predicted deleterious variants in the *NOS1* gene (Figure 7(b) to (d)). The group of individuals with exonic, rare predicted deleterious variants in *NOS1* showed a shorter time in mixed physical activity, and they showed lower standard deviation in duration of the day (Figure 7(e)). Both detected phenotypes support findings in a larger sample of ASPE and SPARK participants examined in our study.

## Discussion

In this study, we combined questionnaire- and accelerometer-based measures to investigate the relationship between sleep, physical activity, and autism features in 262 autistic participants and 130 non-autistic relatives using 3 weeks of actimetry data. For this study, we focused on adults (ages 18–60) to reduce the likelihood of confounding factors due to age and developmental considerations. Also, we recruited participants without ID to alleviate the impact of repetitive movements in autism with ID. We calculated each participant’s mean, median, and standard deviation for 154 actimetry-derived sleep, physical activity, and circadian features. Our data, derived from objective activity-based measured sleep characteristics, reveal less robust differences between autism and its non-autistic relatives compared to self- or parent reports (K. P. Johnson & Zarrinnegar, 2024; Liang et al., 2024; Petruzzelli et al., 2021). For example, there was no significant difference in total sleep duration; however, the variance of sleep duration was significantly higher in the autistic participants than in their family members. Our results indicate that the differences in sleep features in autistic adults without ID may be more subtle between autistic individuals and their non-autistic relatives. In an unbiased approach evaluating all actimetry-derived features, we did, however, identify physical activity traits such as light physical activity and time spent sedentary, in addition to the day-to-day variability of circadian traits such as M10, as different between the groups, where autistic individuals were more sedentary with shorter periods of light physical activity.

To our understanding, this is the largest reported study in autism that examined sleep characteristics from actimetry for at least 3 weeks. Rather than testing specific hypotheses, we used machine learning methods to evaluate 308 total actimetry-derived sleep: wake characteristics, and their potential associations with autism in a high-throughput, data-driven approach. In the best-fit model, measures of both mean and standard deviation values of actimetry-derived traits were selected, suggesting the importance of examining the day-to-day variability and general average differences in sleep and physical activity in autism. The relationship between these selected traits and several measures of sleep quality were also evaluated for potential interaction with autism status. Here, we found that total time spent sedentary had a different relationship with total sleep time in autistic individuals compared to their non-autistic relatives, with autistic individuals having more sleep loss for the same amount of sedentary time than their non-autistic relatives. This may suggest a different relationship between physical activity and sleep in autistic individuals, where physical activity is more strongly tied to sleep in those with autism, a finding with potential treatment implications. Future research to parse out the relationships between physical activity, sleep, and autism is warranted.

Interestingly, many of the traits selected from the elastic net were physical activity rather than sleep, further supporting the importance of the general level or pattern of activity in autism. Several groups reported that deficits in motor skills in autistic children and adolescents can predict social deficits (MacDonald et al., 2013; Pan et al., 2011). Further parallel studies of physical activity and deficits in motor skills are needed to better understand the basis of the overall lower level of activity in autism. The relationship between selected physical activity traits and several measures of sleep quality were also evaluated, considering potential interactions with autism status. Here, we found that total inactivity and total light activity during the day had a stronger correlation with total sleep time in autistic individuals compared to their UR. We observed that autistic individuals had lower sleep regularity compared to their non-autistic relatives. Moreover, our finding that higher sleep regularity in individuals with and without autism is linked to higher physical activity levels provides additional support for increased physical activity as a potential treatment for sleep disturbances in autism (Healy et al., 2018; Rivera et al., 2025). In many conditions with co-occurring sleep disturbances, the association is probably indirect via behavior and properties of the circadian system. The less time affected subjects go outside (often combined with higher physical activity) the lower their daily amplitudes in light and activity (Burns et al., 2023; Roenneberg et al., 2022). Yet, high amplitudes in light and activity contribute to regularity of sleep and activity timing. This also seems to be the case in autism, which opens up therapies based on improved irregularity of sleep–wake cycles.

Our study has several limitations. First, in addition to the need to extend our study to a larger set of both autistic children and adults, we also need an independent set of unrelated non-autistic participants to externally validate our findings and enhance generalizability. Although initially our family-based study included a larger sample of participants, we focused our analyses on adults, ages 18–60, without ID. In a significantly larger-scale study, we would be able to better address age-dependent changes in sleep:wake and physical activity patterns in autistic individuals. Finally, to evaluate both sleep and physical activity in autistic participants, we opted to perform an accelerometer-based study combined with self-reports. Our data showed that a significant number of autistic subjects with regular sleep:wake patterns detected by accelerometer devices initially reported sleep disturbances. Polysomnography (PSG), the gold-standard assessment for sleep/wake disturbances, may help to better understand underlying deficits by electrophysiological evaluation of sleep stages and in the diagnosis of sleep-related breathing or movement disorders missed by actimetry.

An important aspect of our moderate-sized accelerometer-based research study is clinical implications. Currently, clinical assessments of autism and related sleep disturbances rely mostly on informant-reported and self-reported questionnaires. Previously, we reported poor agreement between self- and informant-report measures for autistic traits with significant reporting discrepancies and often self-reporting more autism-related behaviors (Taylor et al., 2024). Here, we report moderate correlations between self-reported total sleep time, sleep onset time, and sleep–wake time and corresponding actimetry-derived measures, yet poor correlation with sleep efficiency, similar to what has been found by other research groups (Zinkhan et al., 2014). This may in part be due to underestimation of the WASO and potentially inaccurate estimation of sleep latency(Valko et al., 2021). Commercially available wearable devices offer affordable and reliable objective monitoring of sleep and activity patterns over prolonged time periods (Babu et al., 2024). Moreover, future clinical assessments and large-scale research investigations of sleep architecture in autism will involve a range of sensors, including simultaneous monitoring of heart rate variability, which has been shown to associate with psychiatric conditions, may provide insights into the autism pathophysiology (Cheng et al., 2020).

Continued longitudinal research into the sleep and activity patterns of autistic children and adults should include the evaluation of day-to-day variability of sleep and physical activity measures, as these appear to be strong indicators of autism status. Previously, researchers have investigated the relationship between physical activity and sleep in the general population, but our findings of an interaction in this relationship with autism support the utility of more research in the autism space. Taken with prior reports showing improvement of autism symptoms after interventions designed to improve physical activity, long-term studies evaluating the relationships between sleep, physical activity, and autism should be conducted to evaluate the potential therapeutic benefits of intervention in these domains.

## Supplemental Material

sj-csv-1-aut-10.1177_13623613251413538 – Supplemental material for Sleep and activity patterns in autism Supplemental material, sj-csv-1-aut-10.1177_13623613251413538 for Sleep and activity patterns in autism by J Dylan Weissenkampen, Arpita Ghorai, Thaise NR Carneiro, Maria Fasolino, Brielle N Gehringer, Maya Rajan, Holly C Dow, Shriya Kunatharaju, Till Roenneberg, Ronnie Sebro, Daniel J Rader, Brendan T Keenan, Laura Almasy, Edward S Brodkin and Maja Bućan in Autism

sj-csv-2-aut-10.1177_13623613251413538 – Supplemental material for Sleep and activity patterns in autism Supplemental material, sj-csv-2-aut-10.1177_13623613251413538 for Sleep and activity patterns in autism by J Dylan Weissenkampen, Arpita Ghorai, Thaise NR Carneiro, Maria Fasolino, Brielle N Gehringer, Maya Rajan, Holly C Dow, Shriya Kunatharaju, Till Roenneberg, Ronnie Sebro, Daniel J Rader, Brendan T Keenan, Laura Almasy, Edward S Brodkin and Maja Bućan in Autism

sj-pdf-3-aut-10.1177_13623613251413538 – Supplemental material for Sleep and activity patterns in autism Supplemental material, sj-pdf-3-aut-10.1177_13623613251413538 for Sleep and activity patterns in autism by J Dylan Weissenkampen, Arpita Ghorai, Thaise NR Carneiro, Maria Fasolino, Brielle N Gehringer, Maya Rajan, Holly C Dow, Shriya Kunatharaju, Till Roenneberg, Ronnie Sebro, Daniel J Rader, Brendan T Keenan, Laura Almasy, Edward S Brodkin and Maja Bućan in Autism

sj-xlsx-4-aut-10.1177_13623613251413538 – Supplemental material for Sleep and activity patterns in autism Supplemental material, sj-xlsx-4-aut-10.1177_13623613251413538 for Sleep and activity patterns in autism by J Dylan Weissenkampen, Arpita Ghorai, Thaise NR Carneiro, Maria Fasolino, Brielle N Gehringer, Maya Rajan, Holly C Dow, Shriya Kunatharaju, Till Roenneberg, Ronnie Sebro, Daniel J Rader, Brendan T Keenan, Laura Almasy, Edward S Brodkin and Maja Bućan in Autism

sj-xlsx-5-aut-10.1177_13623613251413538 – Supplemental material for Sleep and activity patterns in autism Supplemental material, sj-xlsx-5-aut-10.1177_13623613251413538 for Sleep and activity patterns in autism by J Dylan Weissenkampen, Arpita Ghorai, Thaise NR Carneiro, Maria Fasolino, Brielle N Gehringer, Maya Rajan, Holly C Dow, Shriya Kunatharaju, Till Roenneberg, Ronnie Sebro, Daniel J Rader, Brendan T Keenan, Laura Almasy, Edward S Brodkin and Maja Bućan in Autism

sj-xlsx-6-aut-10.1177_13623613251413538 – Supplemental material for Sleep and activity patterns in autism Supplemental material, sj-xlsx-6-aut-10.1177_13623613251413538 for Sleep and activity patterns in autism by J Dylan Weissenkampen, Arpita Ghorai, Thaise NR Carneiro, Maria Fasolino, Brielle N Gehringer, Maya Rajan, Holly C Dow, Shriya Kunatharaju, Till Roenneberg, Ronnie Sebro, Daniel J Rader, Brendan T Keenan, Laura Almasy, Edward S Brodkin and Maja Bućan in Autism

sj-xlsx-7-aut-10.1177_13623613251413538 – Supplemental material for Sleep and activity patterns in autism Supplemental material, sj-xlsx-7-aut-10.1177_13623613251413538 for Sleep and activity patterns in autism by J Dylan Weissenkampen, Arpita Ghorai, Thaise NR Carneiro, Maria Fasolino, Brielle N Gehringer, Maya Rajan, Holly C Dow, Shriya Kunatharaju, Till Roenneberg, Ronnie Sebro, Daniel J Rader, Brendan T Keenan, Laura Almasy, Edward S Brodkin and Maja Bućan in Autism

sj-xlsx-8-aut-10.1177_13623613251413538 – Supplemental material for Sleep and activity patterns in autism Supplemental material, sj-xlsx-8-aut-10.1177_13623613251413538 for Sleep and activity patterns in autism by J Dylan Weissenkampen, Arpita Ghorai, Thaise NR Carneiro, Maria Fasolino, Brielle N Gehringer, Maya Rajan, Holly C Dow, Shriya Kunatharaju, Till Roenneberg, Ronnie Sebro, Daniel J Rader, Brendan T Keenan, Laura Almasy, Edward S Brodkin and Maja Bućan in Autism
