# Supplementary material for: Sleep and activity patterns in autism
Source: Autism. 2026 Feb 5;30(3):767–87. doi: 10.1177/13623613251413538 (PMC12923632; doi:10.1177/13623613251413538)

Index - Supplementary Tables and Figures

Supplementary Table I: List of all actimetry-derived traits from GGIR and Accelerometer.

Supplementary Table II: 52 actimetry-derived traits associated with autism determined by elastic net.

Supplementary Table III: Area under the curve (AUC) using a transitional fully independent sample set.

Supplementary Table IV: Elastic net selected actimetry-derived traits associated with autism in the independent analysis.

Supplementary Table V: best-fitting model performance in common co-occurring psychiatric conditions.

Supplementary Table VI: Effects of seasonality on machine learning-identified actimetry-derived traits.

Supplementary Table VII: Linear mixed-effect regression results.

Supplementary Table VIII: Social Responsiveness Scale (SRS) stepwise regression results.

Supplementary Table IX: Broad Autism Phenotype Questionnaire (BAPQ) stepwise regression results.

Supplementary Table X: Genomic analysis.

Supplementary Figures

Supplementary Figure 1: Distribution of weekend- and weekdays across the even and odd datasets in ASD and non-ASD subjects (A) and distribution of actimetry recordings collected across four seasons in ASD and non-ASD subjects (B) .

Supplementary Figure 2: Determination of the optimal value of regularization parameter alpha in the elastic net model

Supplementary Figure 3: Comparison of GGIR and sleep-diary estimates for four sleep traits.

Supplementary Figure 4: Correlations between all actimetry-derived traits

Supplementary Figure 5: Comparison of seasonality in machine-learning-selected actimetry-derived traits.

Supplementary Figure Legends

Supplementary Figure 1. Distribution of actimetry recordings across days and seasons. (A) Distribution of weekdays and weekends in those with and those without ASD. There was no significant difference between ASD and non ASD individuals for weekdays and weekends, even after splitting even and odd days. No difference in distribution in even or odd datasets ($p > 0.5$). (B) Season of actimetry recording in ASD and non-ASD individuals. Individuals without ASD were more likely to be assessed with actimetry in summer than those with ASD. ASD individuals were more likely to have actimetry measures in the springtime.

Supplementary Figure 2. Determination of the optimal value of regularization parameter alpha in the elastic net model. A) Performance of the model including age and sex as covariates. B) Performance of the model excluding age and sex as covariates. Model performance was evaluated across a range of alpha values to identify the balance between L1 and L2 penalties that maximized predictive accuracy (AUC).

Supplementary Figure 3. Comparison of GGIR and sleep-diary estimates. (Left) Bland-Altman and (Right) Scatterplots of (A) sleep onset, (B) sleep offset, (C) total sleep time (TST), and (D) sleep efficiency (SE). Each dot is one participant grouped by cohort (ASPE = blue, SPARK =pink). (Left) The x-axis is the average of GGIR and Sleep diary (SD); the y-axis is the difference (GGIR − SD). The solid horizontal line marks the mean bias, and the dashed lines are the 95% limits of agreement (LoA = bias ± 1.96·SD); the dotted line at 0 indicates perfect agreement. onset/offset in decimal hours (values may exceed24 to indicate next-day times), TST in hours, and SE in percent. Positive differences mean GGIR > SD (e.g., later times or larger values); negative differences mean GGIR < SD. (Right) X-axis is sleep diary and y-axis is GGIR. The dashed diagonal (y = x) is the line of identity; points close to this line indicate better agreement, points above the line indicate GGIR > SD, and points below indicate GGIR < SD. Dots are colored by cohort (ASPE = blue, SPARK = pink). Each panel reports the Pearson correlation coefficient (r), p-value.

Supplementary Figure 4. Correlations between all actimetry-derived traits. Blue is a higher positive correlation, while red is negative correlations.

Supplementary Figure 5. Comparison of sleep and activity traits with seasonality. Examples of traits with no impact of season or autism status for sleep onset time (A), wakes after sleep onset (B), or sleep in the scotoperiod (C). Examples of traits such as Time sitting or standing (D) and time in mixed physical activity (F) showing significant differences between autistic and non-autistic participants. Seasonality was significantly different for the average light during the most-active 10 hour period (E).

Supplementary Figure 1

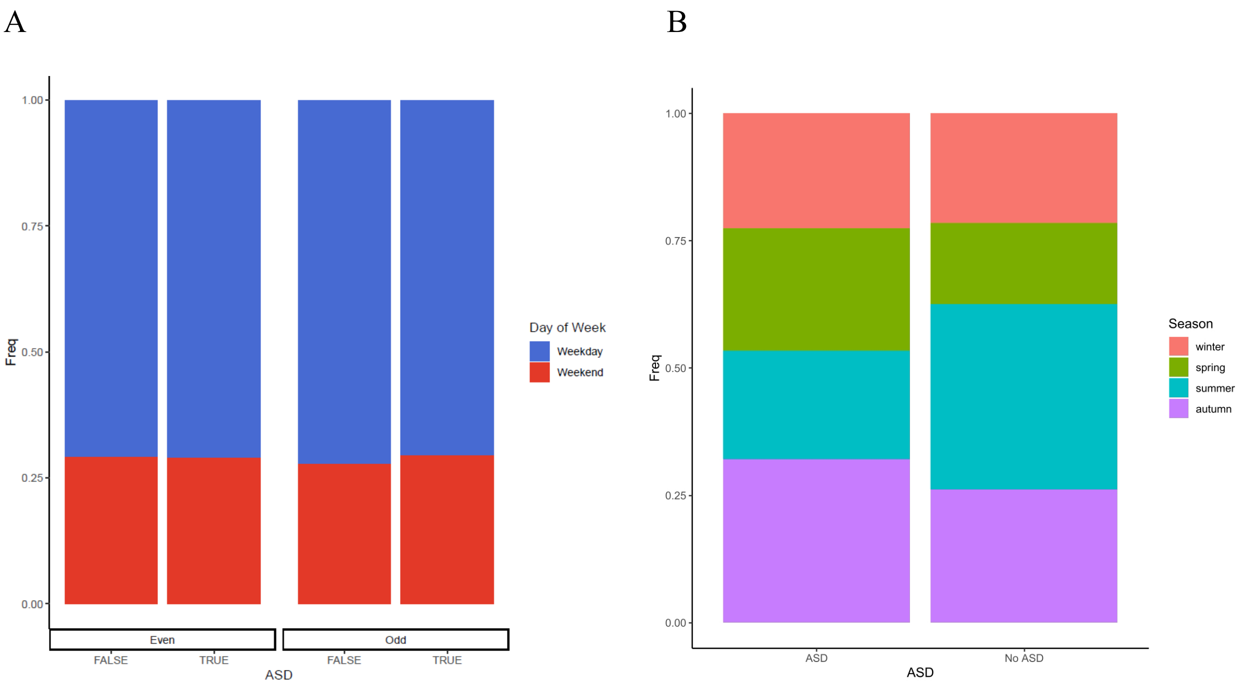

Supplementary Figure 2

A

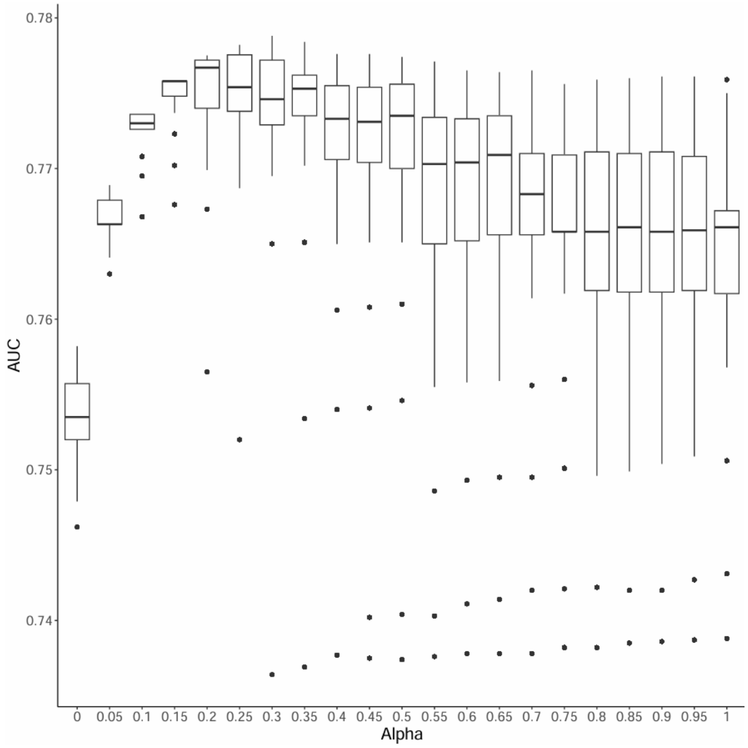

B

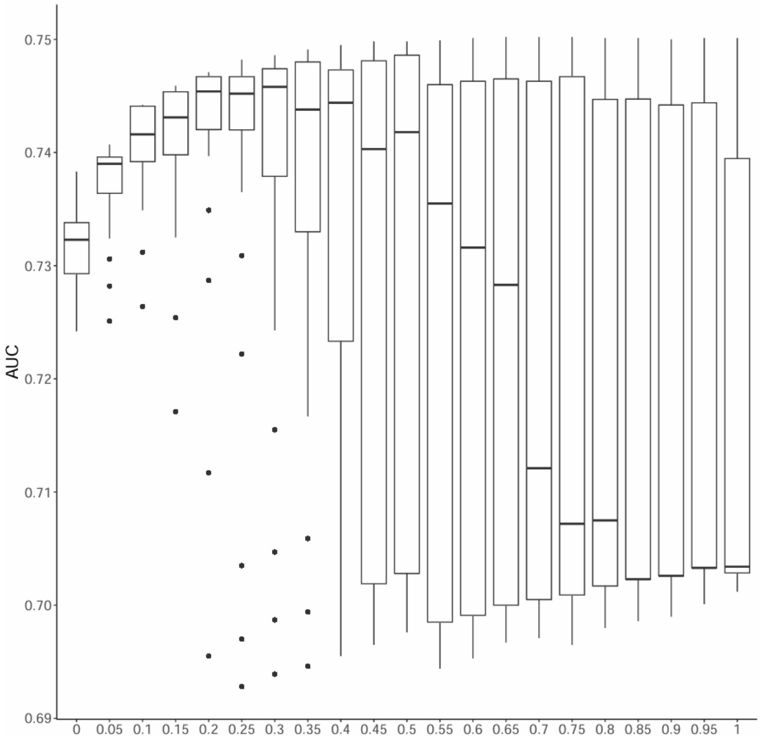

Supplementary Figure 3

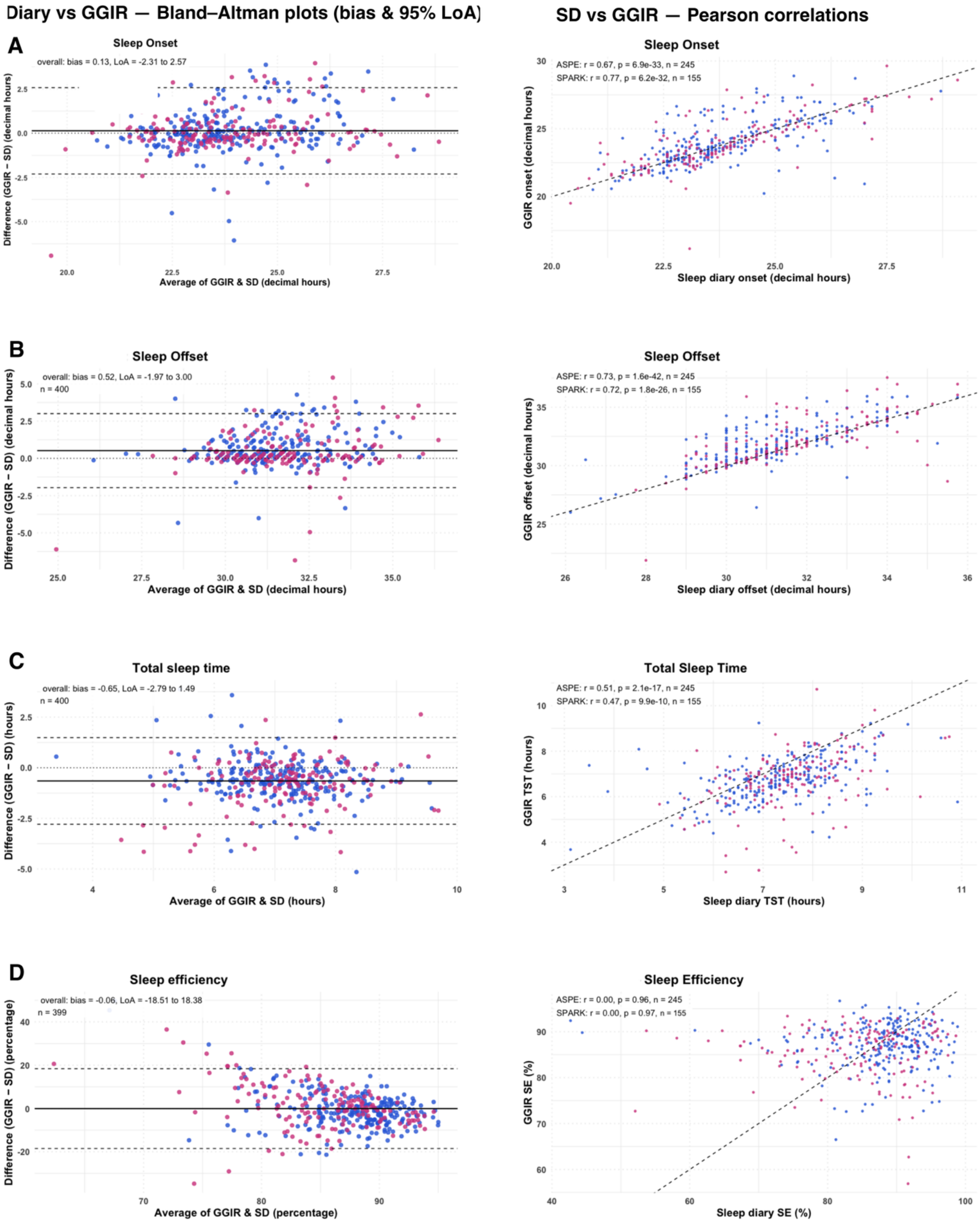

Supplementary Figure 4

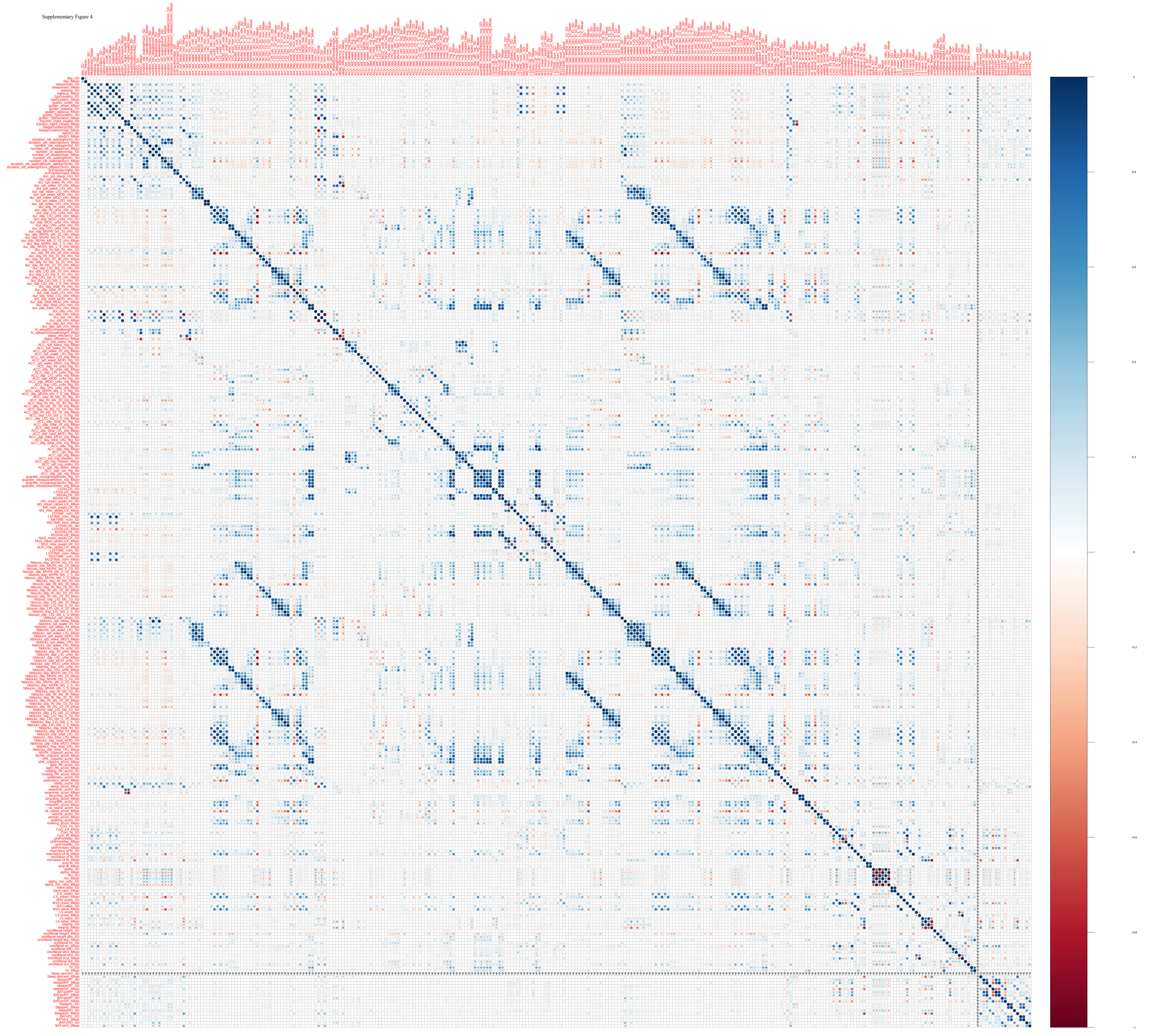

Supplementary Figure 5

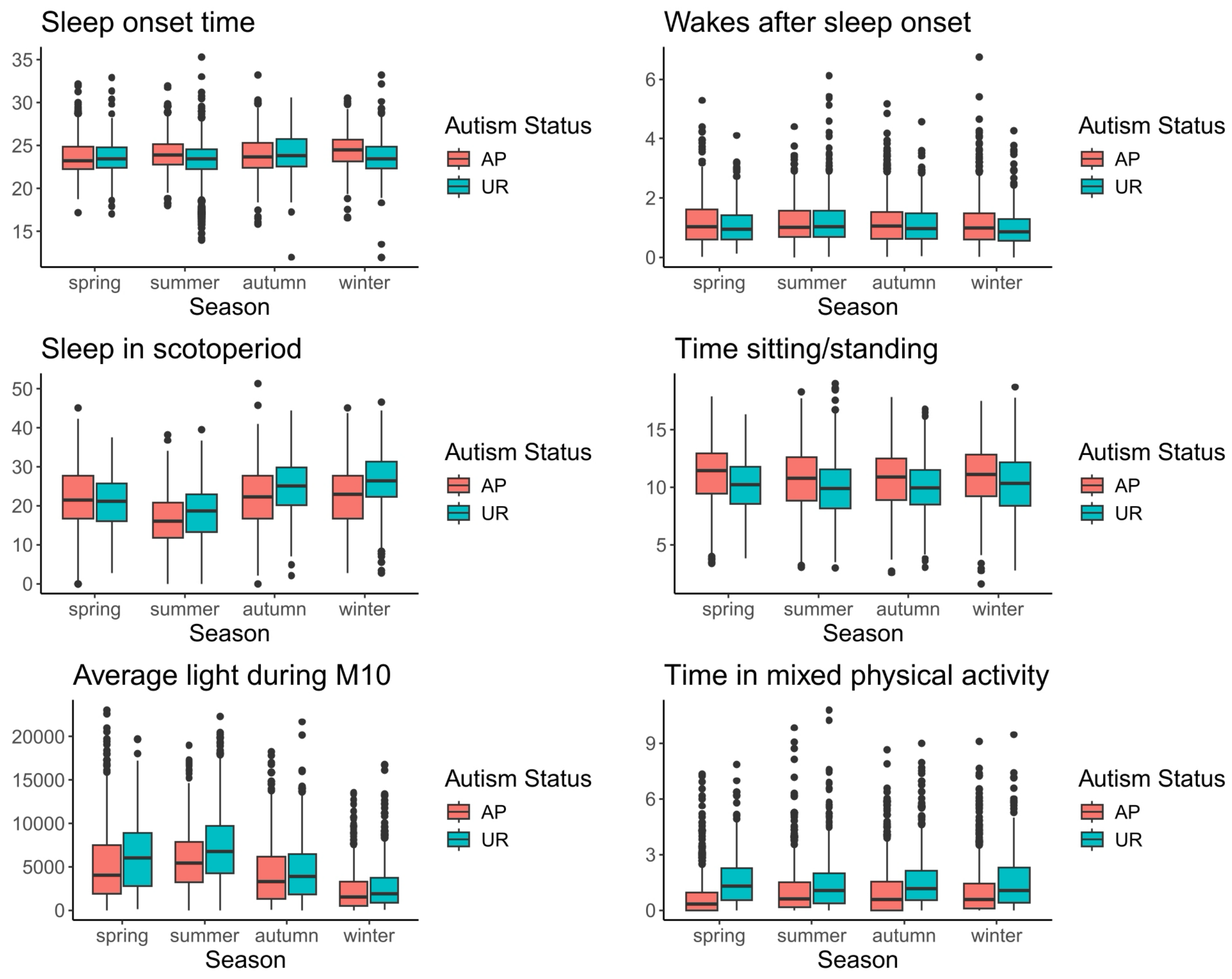

Supplement: sj-pdf-3-aut-10.1177_13623613251413538 – Supplemental material for Sleep and activity patterns in autism [file sj-pdf-3-aut-10.1177_13623613251413538.pdf]
